# Analysis of Bank Credit Risk Evaluation Model Based on BP Neural Network

**DOI:** 10.1155/2022/2724842

**Published:** 2022-03-10

**Authors:** Xiaogang Wang

**Affiliations:** College of Management, Henan University of Technology, Zhengzhou, Henan 450052, China

## Abstract

Commercial banks are of great value to social and economic development. Therefore, how to accurately evaluate their credit risk and establish a credit risk prevention system has important theoretical and practical significance. This paper combines BP neural network with a mutation genetic algorithm, focuses on the credit risk assessment of commercial banks, applies neural network as the main modeling tool of the credit risk assessment of commercial banks, and uses the mutation genetic algorithm to optimize the main parameter combination of neural network, so as to give better play to the efficiency of neural network. After verification of various evaluation models, the accuracy of the evaluation model designed in this paper is more than 65%, while the acceptability of the evaluation results optimized by the mutation genetic algorithm is more than 85%. Compared with the accuracy of about 50% of the traditional credit scoring method, the accuracy of the credit risk evaluation using neural network technology is improved by more than 10%. It is proved that the performance of the optimized algorithm is better than that of the traditional neural network algorithm. It has important theoretical and practical significance for the establishment of the credit risk prevention system of commercial banks.

## 1. Introduction

The credit risk assessment has developed for a long time, and its measurement method is also changing, and the model method has been adopted for the credit risk assessment. The evaluation and management of credit risk is of more important practical significance for commercial banks [1]. The credit risk evaluation of commercial banks is a typical fuzzy problem because there are not only many factors affecting credit risk but also one factor often has multiple levels. Therefore, it is necessary to explore the index system, evaluation method, and practical application of comprehensive evaluation of credit risk. Therefore, it will be of great significance to accelerate the research work of the credit risk assessment, find the credit risk assessment method and model suitable for the actual situation of commercial banks as soon as possible, provide the basis for loan decision making of commercial banks, and finally improve the competitiveness of commercial banks [2]. To solve the problem of the credit risk assessment, neural network technology has its unique advantages in which the most important is the nonlinear mapping ability of neural network. Through the forward neural network, the nonlinear relationship between credit index and rating can be described quantitatively, so as to meet the classification of customer credit rating according to index data [3].

Based on the in-depth study of the current situation and application characteristics of the credit risk assessment of commercial banks, combined with the characteristics of data mining and neural network technology, this paper puts forward a credit risk assessment system [4], improves and verifies the proposed model, and provides a feasible solution for the credit risk assessment. In the assessment system, the core task of data mining is credit risk classification. There are also many data mining methods that can realize classification, such as decision tree and neural network. According to the application characteristics and data characteristics of the credit risk assessment, neural network has better pertinence and applicability than other classification methods. Therefore, neural network method is used to undertake the core task of data mining [5], namely, credit risk classification. However, the collected data cannot be directly used to train the neural network classification model, but must go through certain preprocessing and pattern division. Preprocessing includes supervised discretization, which is to determine split points in a way that maximizes the purity of the interval. However, in practice, this approach may require arbitrary determination of the bin purity and minimized bin size. To address this, some statistics-based methods divide the interval with each attribute value and create larger intervals by merging adjacent intervals similar to those based on statistical tests. The outlier analysis function of data mining is used to find the samples containing outlier data in the training samples, and discard or flatten these samples so that the neural network model can more truly reflect the mapping relationship between credit indicators and credit ratings [6]. In fact, pattern division is also classification. It distinguishes enterprise customers according to their financial data so that the financial data of the same kind of enterprise customers fall within a reasonable range, to improve the classification accuracy of the neural network model [7]. Although BP algorithm has been widely used, the main disadvantage is that the training process is uncertain. The genetic algorithm is a global optimization algorithm. This algorithm is essentially different from the single-point search method. It adopts the method of processing multiple individuals in the search space at the same time. It has good global search performance and effectively reduces the possibility of falling into the local optimal solution [8]. Therefore, the emergence of the genetic algorithm makes the training of neural network have a new look. Using the genetic algorithm instead of BP algorithm to search the connection weight of neural network is expected to solve the problem of BP network falling into local minimum. To sum up, the credit risk assessment system studied in this paper uses neural network to complete the function of the credit risk assessment [9].

In this paper, genetic algorithms are introduced to optimize the network established by backpropagation algorithm to form a hybrid algorithm. For the credit risk, a backpropagation network includes input layer, output layer, and several hidden layers. The principle of the network for the credit risk assessment of commercial banks is to take the index information used to measure the financial and nonfinancial status of loan enterprises as the input vector of God and network, the classification result is used as the output vector of neural network, and the network is trained with training samples to obtain different output values. After the training, it can be used as an effective tool for the credit risk assessment and prediction. After the training, credit risk can be effectively predicted. This paper compares and analyzes the prediction results of neural network optimized by the mutation genetic algorithm to verify its application in the credit risk evaluation of commercial banks. The experimental results show that the mutation genetic algorithm has the ability to optimize the training of neural network, and it is feasible and effective to apply the neural network model optimized by the mutation genetic algorithm to the credit risk evaluation of commercial banks.

## 2. Related Work

The credit risk assessment is the core content of bank risk management. Credit risk will lead to bank bankruptcy. Therefore, since the birth of the bank, banking experts have begun to explore the credit risk assessment [10]. The evaluation of creditability involves many factors, generally including the following five independent and related factors, namely, economic strength, growth ability, asset management ability, solvency ability, and profitability. Since the 1980s, the evaluation of credit risk has changed from relying on the experience and subjective analysis of banking experts to a credit scoring model based on financial indicators, mainly including linear probability model, logit model, probit model, and discriminant analysis model [11]. In the late 1980s, some experts at home and abroad began to use artificial intelligence theory and neural network theory to improve the accuracy and accuracy of distinction. The application of these methods overcame the requirements of the above traditional statistical methods for assumptions and the shortcomings of statically reflecting credit risk. Since the 1990s, the loan profit of commercial banks has continued to decline and the risk of off-balance sheet business has increased, which has prompted banks in various countries to pay more attention to the management and prevention of credit risk. Many Western banks have begun to explore the use of modern financial theory and mathematical tools to develop new credit risk measurement models [12]. They began to develop new and more complex credit evaluation systems, changing from simple single loan risk to measuring centralized risk. All these efforts have made great progress in the measurement and control technology of credit risk in the Western banking industry and produced a series of successful quantitative management models of credit risk. Comprehensive models can be divided into two categories: one is the combination theory model, such as JPMorgan's Credit Metrics and KMV method [13] and the other is the default model, such as credit risk + of Credit Suisse and portfolio view model of McKinsey. The biggest difference between the two models is that the default model estimates the future default distribution of the portfolio based on theoretical analysis, while the portfolio theoretical model estimates the future default distribution of the portfolio from historical data [14].

In the research of the credit risk identification, the most commonly used neural networks are BP neural network [15], RBF neural network, and probabilistic neural network. BP network is a one-way propagation multilayer feedforward network [16]. Due to its good mapping ability to approximate nonlinear functions and parallel processing, it can approximate any nonlinear mapping relationship to obtain the solution of the problem; instead of relying on the prior knowledge and rules of the problem, it has strong adaptability and is very suitable for solving the classification and pattern recognition problems of irregular, multiconstraint, or incomplete data [17, 18]. Due to the perfect theoretical basis and few restrictions that can solve the characteristics of data samples, neural network is widely favored in solving classification problems, especially the test data generation based on genetic algorithm. This method first converts the structural coverage problem into a numerical function optimization problem and then generates the expected test data [19]. The genetic algorithm (GA) was first proposed by American scholar Holland in 1975. It is a computational model simulating Darwin's genetic selection and natural elimination of biological evolution. Its outstanding feature is that it contains steps very similar to biological genetics and evolution [20]. The main advantages of GA are simple, universal, strong robustness, suitable for parallel distributed processing and wide application range [21]. The search performance of genetic algorithm is mainly realized by three operators: selection, crossover, and mutation, especially the mutation operator. It can expand the search range of the algorithm and is more likely to find the global optimal solution. However, due to the wide search range, the convergence speed is affected, so it is very important to design adaptive mutation operation. The traditional genetic algorithm and various improved algorithms use the method of randomly selecting a bit to flip the variable value in the mutation operation, which cannot effectively improve the search performance and stability of binary coding. Variation testing was first proposed by Demilo and Hamlet et al. It is a software testing technology to measure the effectiveness and completeness of test case set [22]. In order to carry out variation test, first, make grammatical small changes to a certain statement of the program to produce a new program, which is called a variant.

## 3. Neural Network Algorithm Optimized by Mutation Genetic Algorithm

### 3.1. Network Structure of Evaluation Model

Neurons and the relationship between them define the structure of neural networks. For the credit risk assessment model, its structure can be expressed by the number of input and output nodes, the number of hidden layers, and the number of nodes in each hidden layer of backpropagation network. In the network structure of the evaluation model, the number of input nodes can be obtained intuitively, which is the number of credit risk evaluation indicators. The number of output nodes of the model can be one or multiple. For the classification model, the number of output nodes is related to the number of classification categories. Assuming that the credit rating is *m*, the number of output nodes of the evaluation model can be *m* or log_2_*m*. In most cases, in order to simplify the structure and improve the training efficiency, a network model with multiple outputs is generally transformed into multiple network models with one output [23]. The structure of each model is the same, but each part is given to a separate model to ensure that the same weights are not applied to the entire image. This paper avoids full weight assignment and keeps weight sharing local; then, each model performs convolution and max pooling and generates some kind of output that outputs a dense layer. Based on this principle, this paper simplifies the output of the evaluation model. Firstly, according to the business needs, there are five levels of credit rating: *A*++, *A*+, *A*, *B*+, and *B*; secondly, referring to the credit scoring method, the output of the evaluation model is converted into a continuous variable. Different value ranges of variables correspond to different credit grades, as shown in Table 1.

In order to evaluate the model, it is very important to determine the reasonable number of hidden layers and the number of nodes in each hidden layer. It is generally believed that increasing the number of hidden layers can reduce the network error and improve the classification accuracy of the evaluation model, but it also makes the model complex, the training time is too long, and it tends to “overfit.”

The error bound of the credit risk assessment model is calculated according to Table 1. Taking class *A*++ credit rating as an example, when calculating the model output error, *A*++ takes 0.95, while when converting the assessment model output to credit rating classification, the value of (0.9, 1.0) interval is mapped to class *A*++, and the maximum difference is used to calculate the output error of the model, and the following can be obtained

(1) $$\begin{array}{c} E=\frac{1}{2}\sum_{k=1}^{l}{\left(d_{k}-y_{k}\right)}^{2}. \end{array}$$

Considering the convergence speed and classification accuracy of the evaluation model, *E*^*P*^=(1/2)(0.85 − 0.8)^2^=0.00125, the error bound *E* of the evaluation model is 0.001. In the process of network training, the error threshold should be predetermined according to the actual situation. The selection of the error threshold is completely determined by the convergence speed of the network model and the learning accuracy of the specific sample. When the *E* value is selected to be small, the learning effect is good, but the convergence speed is slow and the training times increases. The opposite is true if the *E* value is larger.

Generally, when the neural network model enters the later stage of training, the more stable is the connection weight between neurons; at this time, the learning rate should tend to be smaller, because the larger learning rate is easy to cause oscillation in the modification process of weight *W*. At the beginning of training, in order to speed up the convergence of the network, the learning rate is often adjusted to a large value, which needs to change the learning rate dynamically in the learning process. The standard for adjusting the learning rate is whether the error function can be reduced, and the adjustment formula is as follows:

(2) $$\begin{array}{c} \eta_{t+1}=\left\{\begin{array}{cc} \alpha \eta_{t}, & \alpha >1,\\ \beta \eta_{t}, & \beta <1. \end{array}\right. \end{array}$$

Among them, *η* is the learning rate; *t* is the training times; *α*, *β,* and *k* are ratio factors; and *E* is the error function,

(3) $$\begin{array}{c} \eta_{t}=\left\{\begin{array}{cc} \alpha \eta_{t-1}, & E_{t}<E_{t-1},\\ \beta \eta_{t-1}, & E_{t}>kE_{t-1},\\ \eta_{t}, & \text{others,} \end{array}\right. \end{array}$$

and the values in this paper are as follows: *α* = 1.05, *β* = 0.7, and *k* = 1.04.

In order to avoid model training trapped in shallow local minima, momentum term can be considered [24]. The weighted adjustment formula with momentum term is

(4) $$\begin{array}{c} w\left(t+1\right)=w\left(t\right)+\frac{\eta \cdot \partial E}{\partial W_{t}}+\alpha^{2}\Delta \left(t\right), \end{array}$$

where *α* is the momentum factor, and its value is discussed in the previous section of this paper. The effect of introducing momentum term is to make the *η* value change equivalently instead of being a constant value in the learning process, to make the adjustment change toward the average direction of the bottom without large oscillation; that is, momentum plays the role of buffer and smoothing, and finally speeds up the learning speed of the evaluation model.

The momentum adding method is suitable for batch learning. In this way, the error is the sum of the output errors of all training samples, that is,

(5) $$\begin{array}{c} E=\frac{1}{2}\sum_{p=1}^{P}\sum_{k=1}^{l}\left(d_{p,k}^{2}-y_{p,k}^{2}\right). \end{array}$$

Moreover, the error backpropagation is started after the learning of all training samples; that is, the connection weights of each layer are updated. Error backpropagation is that the output error is transmitted back to the input layer by layer through the hidden layer in some form, and the error is allocated to all units of each layer, so as to obtain the error signal of each layer, which is used as the basis for correcting the weight of the unit. The credit risk assessment model optimized adopts the mutation genetic algorithm.

### 3.2. Neural Network Optimized by Mutation Genetic Algorithm

The credit risk assessment system is the core part of credit management information system and the basis for other credit businesses. Its main function is to realize the bank's internal credit rating and provide the basis for other credit businesses and credit risk management. The general structure of the credit risk assessment system consists of three components:

1. Input. Input the index data of credit rating. After preprocessing, the index data are used by the evaluation system. The selection of credit rating indicators plays a very important role in the credit risk assessment, which will be discussed below.
2. Evaluation model. The credit risk assessment model is the core part of the credit risk assessment system. The assessment model reflects the mapping relationship between credit assessment indicators and credit rating, and this relationship can be self-adjusted with the change of environment.
3. Output. From a business perspective, the output is the result of credit rating, that is, credit rating, such as *A*++, *A*+, or *B*+, which can be summarized as classification results. From the perspective of the evaluation model, the output may be a continuous value, which can be converted to the credit rating required by the business after a certain mapping relationship.

Normalization of indicator data refers to scaling the indicator data to make it fall into a small specific interval [25]. The general iterative method is easy to fall into the trap of local minima and the phenomenon of “infinite loop” occurs, which makes the iteration impossible. The genetic algorithm overcomes this shortcoming well and is a global optimization algorithm. Normalization is particularly useful for classification algorithms, such as algorithms involving neural networks or distance metric classification algorithms such as nearest neighbor classification and clustering. For the credit risk assessment model using BP algorithm, standardizing the input values of training samples will help to speed up the learning speed of the assessment model.

The order of the mutation genetic algorithm is selection, crossover, and mutation. We adopt adaptive crossover and mutation probability, as shown in formulas (5) and (6), respectively:

(6) $$\begin{array}{c} p_{c}\left(g\right)=\frac{p_{c}\left(g-1\right)-\left[p_{c}\left(0\right)-0.25\right]}{\max}, \end{array}$$

(7) $$\begin{array}{c} p_{m}\left(g\right)=\frac{p_{m}\left(g-1\right)-\left[0.25-p_{m}\left(0\right)\right]}{\max}, \end{array}$$

where *g* represents the generation algebra, and max is the maximum number of generations.

**Figure 1:**
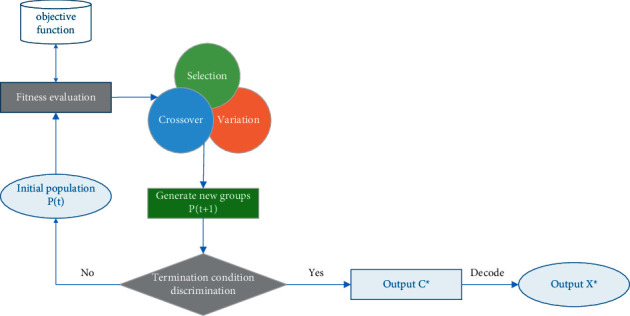
The basic flow of the genetic algorithm.

The basic flow of the mutation genetic algorithm is shown in Figure 1. Similar to the biological evolution process in nature, the operation process of the genetic algorithm is also a repeated iterative process. The population of generation *t* is recorded as *P*(*t*). After one generation of mutation, inheritance, and evolution, the population of generation *t* + 1 is obtained. They are also a population composed of *m* individuals, recorded as *P*(*t*+1). This population continues to undergo mutation, inheritance, and evolutionary operation, and each time, according to the rules of survival of the fittest, more individuals with higher fitness values are inherited to the next generation. In this way, a good individual *c*^*∗*^ will be obtained in the group, and its corresponding phenotype *x* will reach or close to the optimal solution *x*^*∗*^ of the problem.

### 3.3. Implementation of BP Neural Network Algorithm Optimized by Mutation Genetic Algorithm

According to the theoretical analysis, we implement the process of mutation genetic neural network algorithm according to the following steps:

(1) Build hidden layer
  (i) It does not have a fixed formula to apply. It is usually to get a more appropriate value from experience and multiple tests. Through the repeated tests of the test samples in the previous section, the number of nodes in the hidden layer is set at 16.
(2) Coding
  (i) In this experiment, 18 neuron nodes are set in the input layer of the variant genetic neural network as risk prediction, 6 nodes are set in the output layer, the number of hidden layer nodes is 16, and the coding length formula is as follows:
    (8) $$\begin{array}{c} m_{n}=i\cdot m_{i}+j\cdot m_{i}, \end{array}$$
  (ii) i is input layer, *j* is output layer, and mi is hidden layer.
(3) Initialization function
  (i) Here, we initialize through the initializega function. The initialization function used in this paper is initp*p* = initializega (pop, *A*+, “gabpeval”)
(4) Fitness function
  (i) Each chromosome in the genetic algorithm corresponds to a solution of the genetic algorithm. Generally, we use fitness function to measure the advantages and disadvantages of this solution. The formula of fitness function in this paper is
    (9) $$\begin{array}{c} f=k\sum_{j=1}^{n}abs{\left(y_{j},x_{j}\right)}^{-1}, \end{array}$$
  (ii) where *k* is the coefficient, *n* is the number of network output nodes, yi is the expected output, and *x*_*i*_ is the predicted output of the *i*-th node.
(5) Set the parameters of genetic algebra of variation
  (i) Here, the genetic algebra is set at about 100. In the selection operation, choose those with a larger proportion of fitness values to the sum.
(6) Cycle step 2 to step 5 to obtain the simulation results optimized by the mutation genetic algorithm until the training goal is reached or the number of iterations reaches the upper limit goal.

### 3.4. Credit Risk Evaluation System Architecture of Commercial Banks

**Figure 2:**
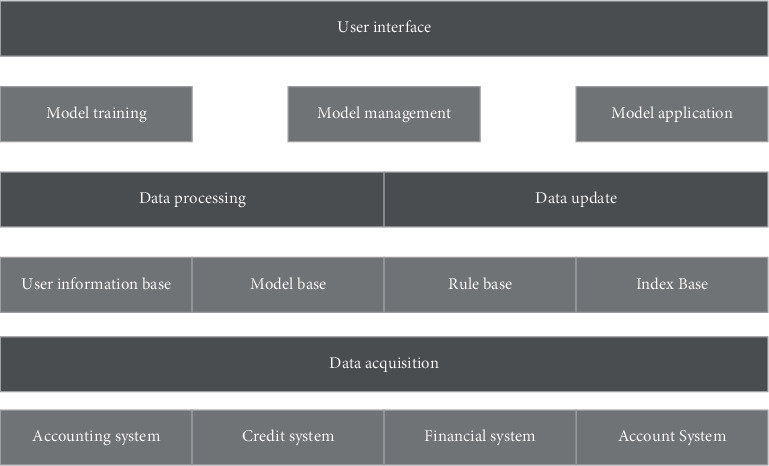
General framework of the credit risk assessment system.

The credit risk assessment system is a data analysis system based on pattern recognition. Firstly, a specific learning algorithm is used to find out the patterned relationship between customer data and credit rating from a large amount of historical data, and then expresses and stores this relationship in the form of the evaluation model for future credit risk evaluation. Therefore, from the perspective of its function, the credit risk assessment system should include the following contents: data collection, data storage, data preprocessing and data maintenance, assessment model training, assessment model management, and assessment model application.  Figure 2 shows the overall architecture of the credit risk assessment system.

Among them, evaluation model training and evaluation model application constitute the core module of the credit risk evaluation system. The main function of the evaluation model training module is to generate and train the model, and store the evaluation model in the model base.

**Figure 3:**
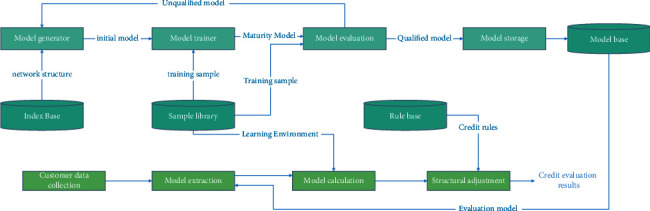
Structure diagram of application module of the credit risk assessment model.

Figure 3 shows the structure of the evaluation model training module. As can be seen from the figure, the model training module is mainly composed of model generation, model training, and model evaluation:

1. Model generation. Model generation first obtains the mode category of the model to be trained (generally classified by industry) from the user interface and then obtains the number of all indexes participating in the evaluation under the mode from the index library. From this number, the number of input neurons and output neurons of the neural network model can be obtained, and the number of neurons in the hidden layer can be calculated. Another main function of the model generator is to initialize the weights or thresholds of the model and learning parameters.
2. Model training. Model training reads out the training samples of the corresponding pattern category from the sample database, processes the data, learns the processed data, and adjusts the weight and threshold of the initial model.
3. Model evaluation. Obtain test samples from the sample library, test the trained evaluation model, and check the evaluation accuracy of the evaluation model. If the accuracy is greater than the accuracy set by the system, the model will be put into storage. Otherwise, discard the model and start training again. The first mock examination is repeated. Sometimes, it is necessary to train the evaluation model of the same pattern type for many times to get the highest accuracy evaluation model.

## 4. Experiment and Analysis

### 4.1. Preparation of Sample Data

A total of 360 data samples were collected in this paper, which were distributed in five industries. After the mode division in the previous section, 360 samples are divided into 7 credit risk assessment modes, and each mode contains more than 30 samples. This section takes industrial enterprises as an example to introduce sample data preparation and preprocessing.

**Figure 4:**
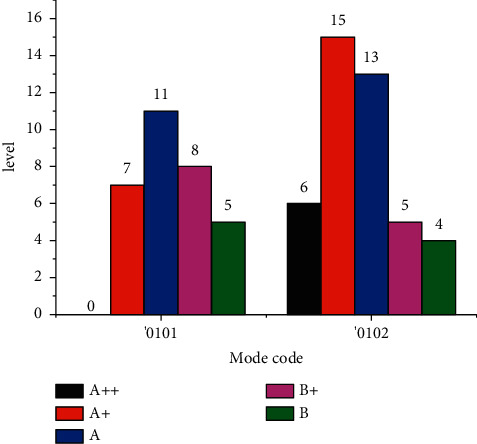
Classification and distribution of credit rating.

Industrial enterprises include two evaluation models: small industrial enterprises and large- and medium-sized industrial enterprises. Among them, small industrial enterprises include 34 samples and large- and medium-sized industrial enterprises include 45 samples.  Figure 4 shows the distribution of credit rating classification in the sample.

**Figure 5:**
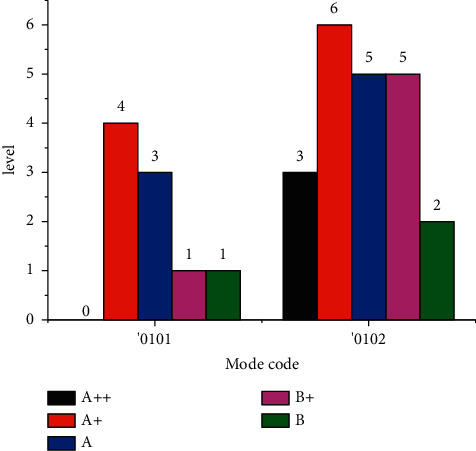
Classification and distribution of credit rating in test samples.

The sample data consist of training samples and test samples. The test samples are randomly selected from the overall samples, generally accounting for about 20% of the total samples. In order to ensure the rationality of the test, this paper requires that each credit rating level contains at least one test sample, and the distribution of credit rating in the test samples is similar to that of the overall samples. The distribution of test samples under the two evaluation modes is shown in Figure 5, while 0101 evaluation mode contains 7 test samples and the remaining 27 samples are used as training samples, and 0102 mode contains 9 test samples and the remaining 36 samples are used as training samples.

Before model training, sample data need to be preprocessed to improve the efficiency of model training and evaluate the accuracy of the model. The main idea of the BP algorithm is to input the learning samples and use the backpropagation algorithm to repeatedly adjust the weights and deviations of the network to make the output vector as close to the expected vector as possible. When the training is completed, the weights and biases of the network are saved. In this paper, the collected qualitative and quantitative indicators of enterprises are standardized, and the index data are transformed into dimensionless data in (0, 1) interval, so as to meet the requirements of input data of variant genetic neural network model. Data processing is divided into two parts: qualitative index processing and quantitative index processing.

### 4.2. Credit Risk Assessment Classification Model

Firstly, the basis and activate function of neuron operation at each node of the evaluation model are selected. According to the general definition of variant genetic neural network model, the basis function of the evaluation model adopts linear function,

(11) $$\begin{array}{c} u=x^{T}w-\theta . \end{array}$$

The activation function adopts sigmoidal function,

(12) $$\begin{array}{c} f\left(u\right)={\left[1+\exp\left(-\lambda u\right)\right]}^{-1}. \end{array}$$

Secondly, the neural network structure of the credit risk assessment classification model is determined. According to the discussion in Section 3.4 of this paper, the evaluation model adopts the variant genetic neural network model with three-layer structure, and its structure can be expressed as *n* × *m* × *l* where *n* is the number of input nodes, and the value is the number of evaluation indicators; *l* is the number of input nodes, and the value is 1; and *m* is the number of hidden layer nodes, and the value is related to many factors. Next, taking the evaluation model of industrial enterprises as an example, the hidden layer node is determined.

The evaluation model structure of industrial enterprise evaluation model is 28 × *m* × 1, and the number of training samples for small- and medium-sized enterprises is 27, and the number of training samples for large- and medium-sized enterprises is 36. According to the rule that the number of hidden layer nodes is between 50% and 70% of the sum of the number of input nodes and output nodes, the value range of the number of hidden layer nodes *m* is about 14 < *m* < 21. In order to determine the reasonable number of hidden layer nodes, this paper constructs an evaluation model for each value within the value range and determines the most reasonable number of hidden layer nodes by comprehensively comparing the training efficiency and classification accuracy of each model.  Table 2 shows the training efficiency and classification accuracy corresponding to the number of nodes in different hidden layers under the same other conditions.

After comparison, we finally selected a three-layer mutation genetic network structure with 16 hidden layer nodes as the evaluation model, and the specific structure is 28 × 16 × 1; that is, the evaluation model includes 28 input nodes, 16 hidden layer nodes, and 1 output node. The learning and calculation process of the evaluation model, for example, K, under this mode can be obtained.

According to the analysis on the setting of learning times, target error, and learning rate in the previous chapters, after establishing the variant genetic neural network with MATLAB software, the initial parameters of these three parameters need to be set before network training on normalized samples, where net.trainparam.epochs is the number of network training, net.trainparam.goal is the network target error, and net.trainparam.lr is the network learning rate. When the initial parameters of the three are set, the train function is called to train the input training samples. The general form of the function is [net, tr] = train (net, *P*, T), net on the left side of brackets represents the genetic neural network before training, tr storage training records, including the number of network training and the error of each training, can generate the network error variation curve in real-time; net on the right represents the mutation genetic neural network obtained after sample training, *T* is the vector set of expected output of training samples, and P is the vector set of training samples.

The procedure is as follows:

- %Set training parameters
- net.trainParam.show = 50
- net.trainParam.epochs = 3000
- net.trainParam.goal = le- 3; net.trainParam.Ir = 0.3
- %Network training
- [net, tr] = train (net, *p*, *t*)

### 4.3. Training Process and Results

In the variant genetic neural network model, there are several very important learning parameters: the main one is the learning rate *η* and momentum factor *α*. The setting of these parameters has a great impact on the training efficiency of the credit risk assessment model. In Section 3.1, it is determined that the value of convergence error bound *e* is 0.001. In order to enhance the convergence efficiency, a large learning rate *η* is usually used, but *η* too large may lead to oscillation near the stable point or even nonconvergence.

**Figure 6:**
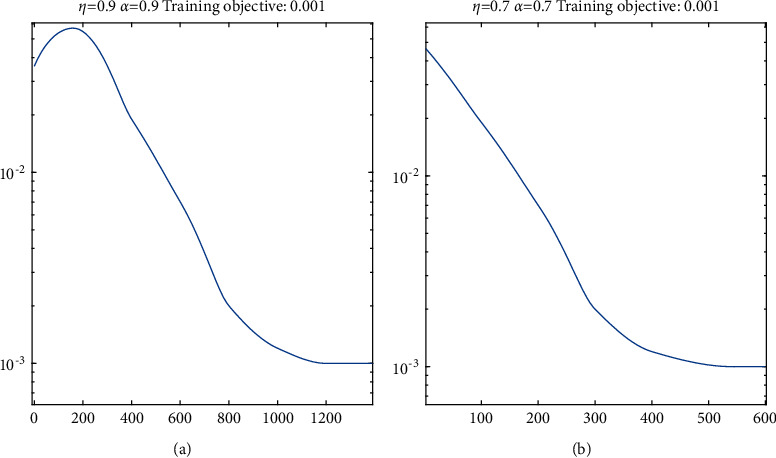
Training error function under different *η* and *α*. (a) Training times: 1178; (b) training times: 544.

For the above situation, this paper analyzes different learning rates *η* and momentum factor *α* simulation investigation is carried out to determine the appropriate learning parameter value. Firstly, 28 are generated by MATLAB neural network toolbox × 18 × 1, and initialize, and then train the network. Set the convergence error bound *e* = 0.001, the maximum training times *T*_max_ = 2000, and select traingdm as the learning function *η* and *α* take values for network training. The network training error curve is shown in Figures 6(a) and 6(b). Figures 6(a) and 6(b) reach the predetermined accuracy within *T*_max_, but the training times are very different when they reach the predetermined accuracy.  Figure 6(a) takes 1178 steps to reach the predetermined accuracy, and Figure 6(b) takes 544 steps to reach the predetermined accuracy.

In the process of network learning, although the initialization of connection weights and thresholds is set randomly, they have a very important impact on the training process of evaluation model, which is embodied in the convergence efficiency and classification accuracy of evaluation model. Under the same conditions, when the initial network weights and thresholds are different, some training processes can converge within the set *T*_max_ range, while some learning processes cannot reach the predetermined accuracy after learning *T*_max_ times. In this case, it is necessary to regenerate the network weights and retrain. In the process of practical application, it is often necessary to initialize the network connection weights and thresholds for many times, and compare the training process and test results for many times to obtain the most reasonable evaluation model.  Table 3 shows the initial weight training and test results of different networks.

### 4.4. Comparative Experiment

**Figure 7:**
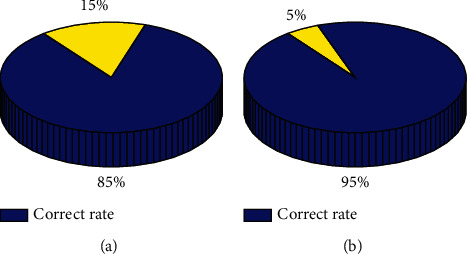
Comparison of training accuracy between the two algorithms. (a) BP algorithm; (b) our algorithm.

Firstly, a simple three-layer mutation genetic neural network structure is used to evaluate credit risk without adding genetic algorithm. Then, the weight and threshold of neural network are optimized by the mutation genetic algorithm. Its operation speed shows greater advantages in speed and quality than BP neural network, and achieves higher accuracy. The training results are shown in Figure 7. Comparing the training time with the original data, Table 4 is obtained. From its fast operation speed and high accuracy, it can be concluded that the variant genetic neural network has strong advantages in the evaluation of credit risk of commercial banks.

**Figure 8:**
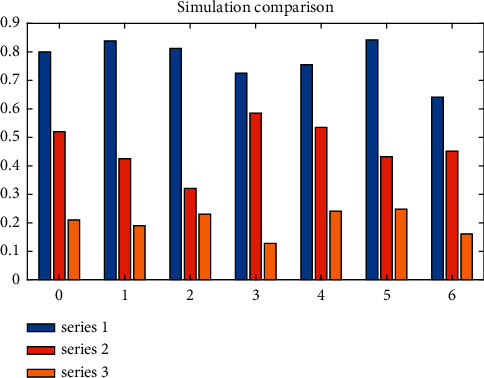
Simulation comparison of evaluation models under two algorithms.

The improved mutation genetic algorithm evaluation model has not only improved the training speed, but also greatly improved the classification accuracy. First select 1000 times to see whether the final training reaches the target error. Then increase or decrease the training times according to the situation. If the training times are the same each time, the degree of training results is very small. It is recommended to increase the number of training times. In the experiment, 2000 times is the best training times. Under the same training steps (set as 2000 steps in this paper), the error accuracy of the basic BP algorithm evaluation model can just reach the accuracy of 0.001, while the error accuracy of the improved mutation genetic algorithm evaluation model can reach 0.0001 at 2000 steps. As shown in Figure 8, the simulation comparison diagram of the evaluation models under the two algorithms is shown, in which series 1 is the expected output, series 2 is the improved algorithm's simulation output, and series 3 is the simulation output of the unmodified algorithm. The number of classification error samples of the evaluation model is 4 by using the unmodified BP algorithm, while the number of classification error samples of the evaluation model is reduced to 2 by using the improved mutation genetic algorithm.

## 5. Conclusion

The credit risk assessment model established in this paper adopts a three-layer mutation genetic neural network structure. For different assessment models, the assessment model has different network structures, which is embodied in the different number of input layer nodes, hidden layer nodes, and their network connection weights. Taking industrial enterprises as an example, the experimental results show that there are two evaluation modes of credit risk: small industrial enterprises and large- and medium-sized industrial enterprises. Their network structure is the same, 28 × 18 × 1. In the learning algorithm, this paper uses the combination of variable learning rate and momentum term to improve the training efficiency and learning rate of the evaluation model *η* value 0.8, momentum factor *α* value 0.9, and error accuracy *ε* set to 0.001. According to the above network structure and learning parameters, the credit risk assessment model is established, and the qualitative index standardized data and quantitative index standardized data are used to train the two assessment models, respectively. Due to the randomness of connection weight initialization, this paper initializes and trains each mode for many times and takes the model with the best test accuracy as the final model. Under the same conditions, the training convergence speed of the improved mutation genetic algorithm evaluation model is very different from that of the basic BP algorithm evaluation model. The training speed of the improved BP algorithm evaluation model has been qualitatively improved. The basic BP algorithm evaluation model takes 1178 steps to meet the accuracy requirements, while the mutation genetic algorithm evaluation model takes only 544 steps to meet the progress requirements. It can be seen that the credit risk assessment model based on neural network has high accuracy and can be used as an important tool for loan decision making of commercial banks.

## Figures and Tables

**Table 1:** Relationship of output of evaluation model and credit rating.

Credit rating	Output of training phase evaluation model
*A*++	(0.9, 1.0]
*A*+	(0.8, 0.9]
*A*	(0.7, 0.8]
*B*+	(0.6, 0.7]
*B*	(0, 0.6]

**Table 2:** Model training under a different number of hidden layer nodes.

Mode code	Number of hidden layer nodes	Simulation convergence
0101	5	Within 2000 training times, the simulation does not converge
	10	>1500 training,
	15	>1300 training,
	20	<1200 training
	25	<1100 training
0102	5	Within 3500 training times, the simulation does not converge
	10	Within 3500 training times, the simulation does not converge
	15	>1800 training
	20	<1800 training
	25	<1600 training

**Table 3:** Model training under different network initial weights.

Mode code	Network weight sequence number	Simulation convergence	Number of classification errors
0101	Initialization 1	Within 3500 training times, the simulation does not converge	15
	Initialization 2	Simulation convergence, training steps 1875	12
	Initialization 3	Simulation convergence, training steps 1168	10
0102	Initialization 4	Simulation convergence, training steps 1050	11
	Initialization 5	Simulation convergence, training steps 978	9
	Initialization 6	Within 3500 training times, the simulation does not converge	11
0103	Initialization 7	Within 3500 training times, the simulation does not converge	14
	Initialization 8	Simulation convergence, training steps 2238	10
	Initialization 9	Within 3500 training times, the simulation does not converge	12

**Table 4:** Comparison of training efficiency between the two algorithms.

	BP algorithm	Our algorithm (s)
Training time	7.230 s	4.836

## Data Availability

The data used to support the findings of this study are available from the corresponding author upon request.
